# Cognitive Impairment Across the Spectrum of Chronic Kidney Disease: Clinical Characteristics and Implications for Practice—Is There a Role for Medical Nutritional Therapy?

**DOI:** 10.3390/nu18183035

**Published:** 2026-09-16

**Authors:** Mihaela Neicu, Constantin Verzan, Adrian Dusa, Elena Stepan, Cristian Iuliu Mihail Iorga, Geanina Beldea, Sebastian Simtea, Liliana Garneata

**Affiliations:** 1 Clinical Hospital of Nephrology “Dr. Carol Davila”, 010731 Bucharest, Romania; 2 Faculty of Sociology and Social Work, University of Bucharest, 050663 Bucharest, Romania; 3 Department of Nephrology and Dialysis, “Sf Ioan” Clinical Emergency Hospital, “Carol Davila” University of Medicine and Pharmacy, 050474 Bucharest, Romania; 4 Department of Nephrology, “Dr Carol Davila” Teaching Hospital of Nephrology, “Carol Davila” University of Medicine and Pharmacy, 050474 Bucharest, Romania; 5 Department of Surgery, “Dr Carol Davila” Teaching Hospital of Nephrology, “Carol Davila” University of Medicine and Pharmacy, 050474 Bucharest, Romania

**Keywords:** chronic kidney disease, cognitive impairment, executive dysfunction, dietary intervention, neuro-cognitive screening

## Abstract

Cognitive impairment is increasingly recognized as a frequent and clinically significant complication of chronic kidney disease (CKD). Across the CKD spectrum, patients commonly exhibit a cognitive profile dominated by executive dysfunction and slowed processing speed, reflecting cumulative vascular, metabolic, and systemic burden. Cognitive impairment is not merely an epiphenomenon of aging but a modifier of clinical trajectory, with implications for treatment adherence, decision-making capacity, dialysis planning, transplantation, and mortality risk. This narrative review synthesizes current evidence regarding epidemiology, domain-specific cognitive patterns, risk modifiers, nutritional determinants, and clinical consequences across predialysis and dialysis populations. We discuss executive–attentional dysfunction and the interaction among kidney dysfunction, vascular burden, depressive symptoms, nutritional status, and cognitive vulnerability. Particular emphasis is placed on pragmatic cognitive assessment and potentially modifiable contributors, including anemia, nutritional deficiencies, dietary patterns, medication-related effects, and dialysis-related hemodynamic instability. Brief screening approaches combining global and executive-function tools may facilitate early identification of clinically meaningful impairment. Integrating cognitive and nutritional assessment into routine CKD care may improve risk stratification, individualized management, and clinical decision-making while identifying potential targets for prevention and intervention.

## 1. Introduction

Chronic kidney disease (CKD) is a major global health burden associated not only with cardiovascular and metabolic complications but also with cognitive impairment [1,2]. Meta-analytic evidence shows that reduced kidney function significantly increases the risk of cognitive decline compared with preserved renal function [3]. Conceptual models further support a kidney–brain axis, reflecting shared vascular and metabolic pathways that may contribute to cerebral vulnerability in CKD [4].

Cognitive impairment is particularly prevalent in dialysis populations, where a substantial proportion of maintenance hemodialysis patients exhibit measurable deficits [5]. Importantly, impaired cognitive performance has been associated with adverse outcomes, including increased all-cause mortality [6].

Despite growing recognition of this association, the clinical cognitive phenotype of CKD and its implications for nephrology practice remain incompletely integrated. This narrative review synthesizes current evidence on the epidemiology and domain-specific cognitive profile of CKD, with particular emphasis on nutritional determinants and other potentially modifiable factors contributing to cognitive vulnerability, as well as their implications for screening and clinical management across the CKD spectrum.

Clinically, cognitive impairment in CKD typically presents not as an overt, easily recognized amnestic syndrome, but as subtle slowing of thought and difficulty with planning, multitasking, and complex decision-making—features that are easily overlooked in routine nephrology visits and may be mistaken for fatigue, inattention, or the effects of comorbid illness. Detection therefore relies on screening instruments rather than clinical impression alone. Commonly used tools include brief global measures such as the Mini-Mental State Examination (MMSE) and the Montreal Cognitive Assessment (MoCA), and executive-function measures such as the Trail Making Test (TMT) Parts A and B; these instruments, along with their comparative strengths and limitations in the CKD population, are discussed in detail in Section 3 and Section 5.

A structured but non-systematic approach was used to identify the relevant literature. PubMed/MEDLINE was searched for articles published within the last 10 years, using combinations of the terms “chronic kidney disease”, “cognitive impairment”, “executive dysfunction”, “dialysis”, “nutrition”, and “medical nutrition therapy”. Articles were selected based on thematic relevance to the epidemiology, clinical phenotype, risk modifiers, screening, and nutritional determinants of cognitive impairment in CKD; reference lists of key articles were also screened for additional relevant sources. As this is a narrative rather than a systematic review, formal PRISMA methodology was not applied.

As a narrative rather than a systematic review, this approach does not include a formal risk-of-bias assessment or a pre-registered protocol, and study selection—while thematically guided—retains an element of author judgment. This approach was chosen to allow a broader, more clinically oriented synthesis across epidemiology, screening, and nutrition, at the expense of the reproducibility and exhaustiveness of a systematic review.

Unlike prior reviews, which have generally addressed either the cognitive phenotype of CKD or its nutritional determinants in isolation, this review integrates both strands into a single clinically oriented synthesis: it links the predominant executive-attentional cognitive profile of CKD to a pragmatic, dual-test screening strategy for nephrology practice, and extends this framework to the nutritional and gut–kidney–brain mechanisms—including protein-energy wasting, uremic toxin generation, and micronutrient balance—that may modify cognitive vulnerability alongside vascular and metabolic contributors. This combined cognitive-and-nutritional lens is intended to support a more comprehensive, actionable approach to cognitive risk in CKD than has typically been offered in the existing literature.

## 2. Epidemiology and Clinical Burden

### 2.1. Prevalence and Risk of Cognitive Impairment Across CKD Stages

Cognitive impairment is prevalent across the CKD spectrum, including predialysis stages. In the CKD-REIN cohort (*n* = 3033; stages 3–4), 13.0% of participants had baseline MMSE scores < 24, and lower estimated glomerular filtration rate (eGFR) independently predicted incident cognitive outcomes (adjusted HR 1.35 per 10 mL/min/1.73 m^2^ lower eGFR; 95% CI 1.07–1.70) [7].

In the CRIC study (*n* = 3883; median follow-up 6.1 years), cognitive impairment defined by the Modified Mini-Mental State Examination (3MS) was present in 13.5% at baseline but was not independently associated with progression to end-stage renal disease (ESKD) or significant decline in kidney function [8].

The burden appears substantially higher in maintenance hemodialysis. In the multicenter COGNITIVE-HD study (*n* = 676), domain-specific impairment ranged from 31.5% to 48.8%, with fewer than one-third of patients demonstrating preserved performance across domains [9].

A meta-analysis of 148 studies confirmed lower global cognitive performance in predialysis CKD and dialysis populations compared with controls, with reduced MMSE scores and prolonged Trail Making Test A completion times across kidney replacement therapy modalities [10].

Across these studies, cognitive status was consistently assessed using validated, standardized instruments—including the MMSE, the 3MS, the MoCA, and structured domain-specific batteries—supporting the comparability of findings despite differences in study design and population.

### 2.2. Clinical Consequences and Prognostic Impact

Cognitive impairment in hemodialysis is associated with adverse outcomes. In a longitudinal cohort of 242 patients followed for a median of 3.54 years, baseline cognitive impairment (MoCA ≤ 24) independently predicted all-cause mortality (adjusted HR 1.749; 95% CI 1.007–3.038) [11].

At the population level, CHARLS data (*n* = 16,515; median follow-up 7 years) demonstrated a higher incidence of cognitive impairment among individuals with CKD (11.46 vs. 6.38 per 1000 person-years), with CKD remaining independently associated after adjustment (adjusted HR 1.56; 95% CI 1.19–2.04) [12].

Collectively, these findings indicate that cognitive impairment in CKD is not only prevalent but also prognostically relevant, being independently associated with incident decline and mortality. Key epidemiological studies are summarized in Table 1.

**Table 1:** Key epidemiological studies linking chronic kidney disease and cognitive impairment.

Ref.	Population (*n*)	Design	Main Finding
[7] (CKD-REIN)	CKD stages 3–4 (*n* = 3033)	Longitudinal	Lower eGFR independently associated with incident cognitive outcomes.
[8] (CRIC)	Mild–moderate CKD (*n* = 3883)	Prospective	Cognitive impairment prevalent; not independently associated with ESKD progression.
[9] (COGNITIVE-HD)	Maintenance hemodialysis (*n* = 676)	Cross-sectional	High prevalence of domain-specific impairment; <30% cognitively intact.
[10]	CKD and KRT populations (148 studies)	Meta-analysis	Lower global cognitive performance and slower executive processing vs. non-CKD controls.
[11]	Maintenance hemodialysis (*n* = 242)	Longitudinal	Baseline cognitive impairment independently predicts all-cause mortality.
[12] (CHARLS)	General population ≥ 45 years (*n* = 16,515)	Longitudinal	CKD independently associated with incident cognitive impairment.

Notes: CKD, chronic kidney disease; KRT, kidney replacement therapy; eGFR, estimated glomerular filtration rate; ESKD, end-stage kidney disease.

## 3. Clinical Cognitive Profile in CKD

### 3.1. Executive Dysfunction and Slowed Processing Speed

Cognitive impairment in CKD is characterized predominantly by executive dysfunction and slowed processing speed rather than isolated amnestic features. In NHANES 2011–2014, stages 3–5 CKD were independently associated with poorer performance on the Digit Symbol Substitution Test (DSST), reduced verbal fluency, and lower immediate recall [13]. A separate NHANES analysis confirmed global impairment with preferential involvement of executive function and processing speed, while delayed recall remained relatively preserved [14].

In maintenance hemodialysis, executive and attentional deficits are even more pronounced. The COGNITIVE-HD study demonstrated frequent impairment in executive and complex attention domains [9], and meta-analytic data confirmed prolonged Trail Making Test completion times and lower global cognitive scores across predialysis and dialysis populations compared with controls [10].

Together, these findings define a predominantly executive–attentional phenotype present in early CKD and accentuated in dialysis.

### 3.2. Memory Profile

Although memory impairment is reported in CKD, the pattern differs from a primary amnestic syndrome. Immediate recall may be reduced alongside executive deficits [13,14], but delayed recall is not consistently associated with reduced kidney function after adjustment [14]. This relative preservation of consolidation suggests that memory deficits in CKD are largely secondary to executive and attentional dysfunction rather than reflecting classical Alzheimer-type pathology.

### 3.3. Dialysis-Related Fluctuations

Cognitive performance in hemodialysis may fluctuate around dialysis sessions. In a prospective study of 28 CKD5D patients tested before and after dialysis, patients performed worse than controls across attention, executive function, and psychomotor speed [15]. A single dialysis session was associated with short-term improvements in several domains.

These findings suggest that, in addition to chronic deficits, a partially reversible component of dysfunction may exist in dialysis patients, highlighting the importance of considering test timing relative to dialysis.

### 3.4. Depressive Symptoms as a Challenge

Depressive symptoms are common in CKD and independently associated with cognitive decline. In NHANES, both depressive symptoms and reduced eGFR were independently linked to cognitive decline, with markedly higher risk when both were present [16].

In hemodialysis populations, depressive symptoms were selectively associated with poorer processing speed and attention but not with global cognition or memory [17]. Peritoneal dialysis represents a distinct treatment setting, with its own spectrum of treatment-related burdens—including mechanical complications such as abdominal hernias [18]—that, alongside psychosocial and treatment-adherence demands, may further contribute to overall patient vulnerability. Thus, depression does not fully account for CKD-related cognitive impairment but may selectively exacerbate executive–attentional dysfunction, underscoring the need for mood assessment during screening.

### 3.5. Differential Considerations

The CKD cognitive phenotype differs from the classical amnestic presentation of Alzheimer-type dementia. Executive dysfunction and slowed processing speed predominate, whereas delayed recall is relatively preserved [14]. In dialysis populations, impairment remains centered on executive and attentional domains [9]. Dialysis-related fluctuations and depression-associated executive deficits further support a predominantly frontal-subcortical (vascular-like) pattern rather than a primary hippocampal syndrome [15,17].

Overall, CKD-related cognitive impairment more closely resembles a vascular or subcortical profile, although mixed mechanisms may coexist.

### 3.6. Cognitive Impairment by Dialysis Modality: Peritoneal Dialysis Versus Hemodialysis

Evidence comparing cognitive outcomes between peritoneal dialysis (PD) and hemodialysis (HD) remains heterogeneous, with no consistent direction of effect. Some studies suggest a possible advantage for PD: a large USRDS-based cohort found that patients initiating PD had a 25% lower risk of incident dementia diagnosis than those initiating HD [19], and a recent systematic review and meta-analysis of 19 studies reported a non-significant trend toward lower cognitive impairment risk in PD compared with HD [20]. Conversely, other studies have found no independent effect of dialysis modality once competing mortality risk is accounted for [21], or have even reported worse cognitive performance in PD than HD [22]. Early cross-sectional data suggest that, although overall burden may be similar between modalities, the domain-specific pattern may differ, with relatively more memory involvement in PD and more executive involvement in HD [23]. This heterogeneity likely reflects differences in study design, cognitive instruments used, and selection bias, since PD is often preferentially offered to younger, healthier patients with fewer comorbidities—a form of confounding by indication that complicates modality comparisons. Well-designed prospective studies with standardized cognitive assessment are needed before firm conclusions can be drawn regarding a protective or detrimental effect of either modality on cognition. Beyond cognitive outcomes, PD carries its own distinct spectrum of treatment-related burdens—including mechanical complications such as abdominal hernias [18]—that may independently influence patient wellbeing and should be weighed alongside cognitive considerations when comparing dialysis modalities.

## 4. Risk Modifiers and Vulnerability

### 4.1. Severity of Kidney Dysfunction (eGFR and Albuminuria)

Severity of kidney dysfunction is a key modifier of cognitive risk in CKD. Reduced eGFR has been consistently associated with poorer executive and processing-speed performance, and longitudinal data demonstrate that declining renal function predicts incident cognitive impairment [12,14].

Albuminuria may confer additional risk beyond reduced eGFR. In population-based data, albuminuria independently predicted future cognitive decline after adjustment for vascular factors [24], suggesting underlying microvascular injury.

Reduced eGFR may also amplify other vulnerability factors. In NHANES, the coexistence of reduced eGFR and depressive symptoms was associated with markedly increased cognitive risk [16].

Together, reduced eGFR and albuminuria identify patients at heightened cognitive vulnerability.

### 4.2. Comorbidities and Systemic Vascular Burden

Cognitive impairment in CKD frequently reflects cumulative vascular and metabolic burden. In CRIC, diabetes, hypertension, and cardiovascular disease were independently associated with worse cognitive performance, even after adjustment for kidney function [25].

Anemia has been linked to lower baseline cognitive scores, although its independent association with longitudinal decline appears attenuated [26], likely reflecting overall disease severity.

In patients with type 2 diabetes and elevated cardiovascular risk, markers of kidney dysfunction were associated with poorer cognition and greater decline over time [27].

Overall, cognitive impairment in CKD appears to arise from interacting vascular and systemic comorbidities rather than kidney dysfunction alone.

### 4.3. Treatment- and Patient-Level Factors

Treatment- and patient-level factors further modulate cognitive vulnerability. In CKD-REIN, exposure to anticholinergic medications was common and independently associated with cognitive impairment [28], highlighting medication burden as a potentially modifiable contributor.

Commonly implicated agents in this population include first-generation antihistamines (e.g., diphenhydramine, hydroxyzine), tricyclic antidepressants, certain antipsychotics, bladder antimuscarinics (e.g., oxybutynin), and some antiemetics and gastrointestinal antispasmodics—several of which are frequently prescribed for symptom management in CKD and dialysis patients.

Dialysis-related hemodynamic instability has been linked to transient and possibly cumulative impairment, particularly in attention and processing speed [15].

Depressive symptoms independently affect executive and processing-speed domains in both predialysis and dialysis populations [16,17]. Educational attainment and cognitive reserve may influence clinical expression.

**Figure 1:**
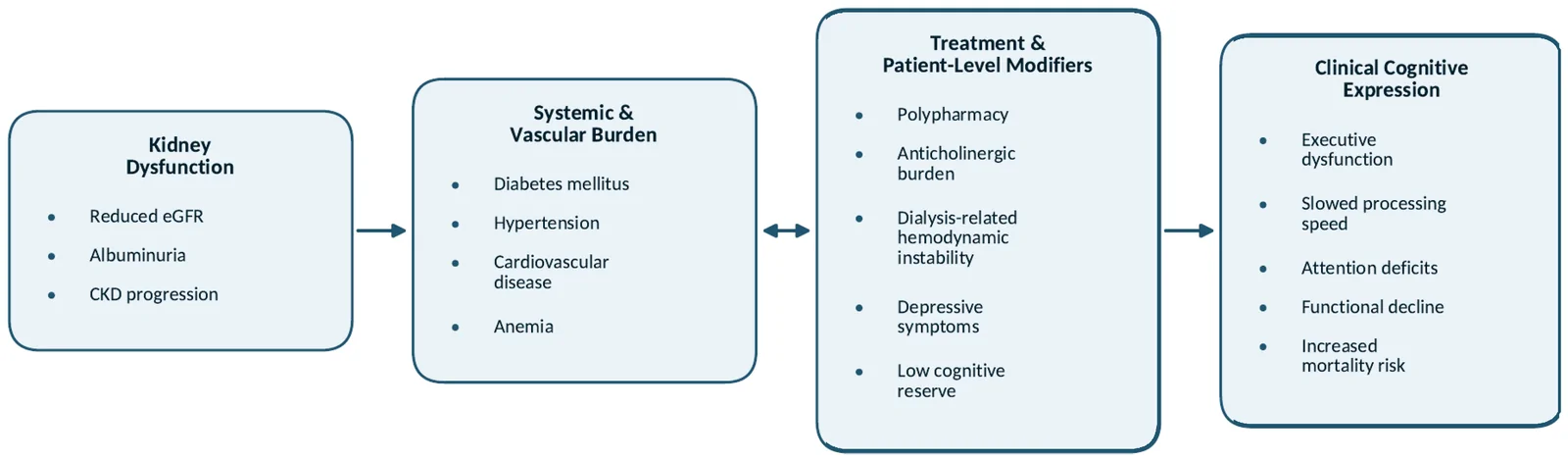
Conceptual framework illustrating the interacting determinants of cognitive vulnerability in chronic kidney disease (CKD). Kidney dysfunction contributes to systemic vascular burden, which dynamically interacts with treatment-related and patient-level modifiers. Together, these factors shape the clinical cognitive phenotype observed in CKD populations.

Medication burden, hemodynamic stress, and mood symptoms therefore shape the clinical manifestation of cognitive impairment in CKD. This conceptual framework is illustrated in Figure 1.

## 5. Practical Screening in Nephrology Practice

### 5.1. Who and When to Screen?

Cognitive impairment is prevalent across the spectrum of chronic kidney disease (CKD), including non-dialysis stages. Data from the CKD-REIN cohort indicate that CKD is associated with a structured cognitive pattern, with preferential executive involvement and longitudinal deterioration over time [29].

Importantly, cognitive deficits are frequently subclinical. In patients with CKD stages 3–5 not yet on dialysis, subclinical impairment has been associated with frailty, reduced mobility, and lower health-related quality of life [30], indicating early functional relevance.

In dialysis populations, cognitive dysfunction is both prevalent and prognostically significant. Lower cognitive performance has been independently associated with increased all-cause mortality in maintenance hemodialysis patients [6]. Depressive symptoms—common in this setting—are additionally linked to poorer executive and processing-speed performance [17].

Despite these data, routine cognitive screening remains uncommon in nephrology practice, largely due to time constraints and uncertainty regarding optimal tools. However, given its impact on adherence, shared decision-making, dialysis modality selection, and prognosis, cognitive assessment should be integrated into comprehensive risk stratification.

Screening does not require extensive neuropsychological batteries. Brief instruments targeting global cognition and executive function are sufficient for first-line identification. The objective is not to diagnose dementia, but to detect clinically meaningful cognitive vulnerability that may influence therapeutic decisions and longitudinal care planning.

### 5.2. Practical Screening Strategy: MMSE, MoCA, and TMT A/B

A pragmatic cognitive screening strategy in nephrology must balance feasibility, time constraints, and adequate domain coverage. Extensive neuropsychological batteries are neither practical nor necessary for routine care. Brief tools targeting global cognition and executive function are sufficient for first-line identification of clinically meaningful impairment [6].

The Mini-Mental State Examination (MMSE) remains the most widely used global screening tool in CKD, both in observational cohorts and meta-analyses [10]. MMSE-based assessments have been used to characterize longitudinal cognitive trajectories in CKD stages 3–4 [29]. However, MMSE alone has limited sensitivity for subtle executive dysfunction, which frequently precedes overt global decline in CKD [17,30].

Executive dysfunction—manifested by slowed processing speed and impaired cognitive flexibility—is a core feature of the CKD cognitive phenotype [17]. The Trail Making Test (TMT) Parts A and B therefore represents a key complement to MMSE, assessing processing speed (TMT-A) and executive control/set-shifting (TMT-B). TMT A/B is consistently among the most frequently applied executive measures across CKD studies [10].

The Montreal Cognitive Assessment (MoCA) has demonstrated greater sensitivity than MMSE for mild cognitive impairment in older adults [31]. Nevertheless, its routine use may be constrained by local validation considerations and the need for longitudinal consistency. A combined MMSE + TMT A/B approach ensures comparability with existing CKD literature while adequately capturing executive vulnerability.

This advantage may be particularly relevant in early-stage CKD (stages 3–4), where cognitive impairment is more likely to be subtle and confined to the executive domain, and where MMSE’s ceiling effects can mask deficits that MoCA is more likely to capture.

Accordingly, a dual-test strategy—MMSE for global cognition and TMT A/B for executive function—provides a feasible and clinically meaningful screening model for nephrology practice [6,10,17].

### 5.3. Proposed Clinical Algorithm

It should be emphasized that the algorithm below is a pragmatic synthesis derived from the reviewed literature and expert clinical reasoning, rather than a prospectively validated clinical guideline; it is intended to support first-line identification of cognitive vulnerability and to prompt further evaluation, not to replace formal neuropsychological or diagnostic assessment.

We propose a pragmatic, stepwise approach to cognitive screening in nephrology. The objective is not to establish a formal dementia diagnosis, but to identify clinically relevant cognitive vulnerability that may influence adherence, shared decision-making, and longitudinal care planning.

Screening should be prioritized in higher-risk patients, including those with CKD stage ≥ 3b, rapid renal decline, high vascular burden, polypharmacy, depressive symptoms, frailty, or functional deterioration. Assessment is particularly relevant before dialysis initiation and during transplant evaluation, when cognitive capacity directly affects complex therapeutic decisions.

A first-line evaluation may combine the Mini-Mental State Examination (MMSE) with the Trail Making Test (TMT) Parts A and B. MMSE provides a global overview, whereas TMT—especially Part B—captures executive dysfunction, a core feature of the CKD cognitive phenotype. This dual-test strategy offers broad domain coverage within a short administration time.

Interpretation should account for age and educational background. Normal results warrant periodic reassessment in progressive CKD. Borderline or isolated executive dysfunction should prompt review of potentially reversible contributors, including anemia, medication burden (particularly anticholinergic load), metabolic disturbances, depressive symptoms, and dialysis-related hemodynamic instability. Global impairment should trigger further evaluation and, when appropriate, referral for specialized assessment.

In dialysis patients, cognitive test timing relative to the dialysis session should be documented, and, where feasible, standardized (e.g., consistently testing before a session or on a non-dialysis day), given evidence that performance may fluctuate acutely around dialysis. Failure to account for this timing may introduce variability that complicates interpretation of borderline results and comparison across visits.

Importantly, screening results must be integrated into routine management. Identification of impairment may justify medication simplification, tailored education strategies, caregiver involvement, and closer adherence monitoring. In this framework, cognitive assessment becomes part of comprehensive risk stratification rather than an isolated diagnostic exercise.

**Figure 2:**
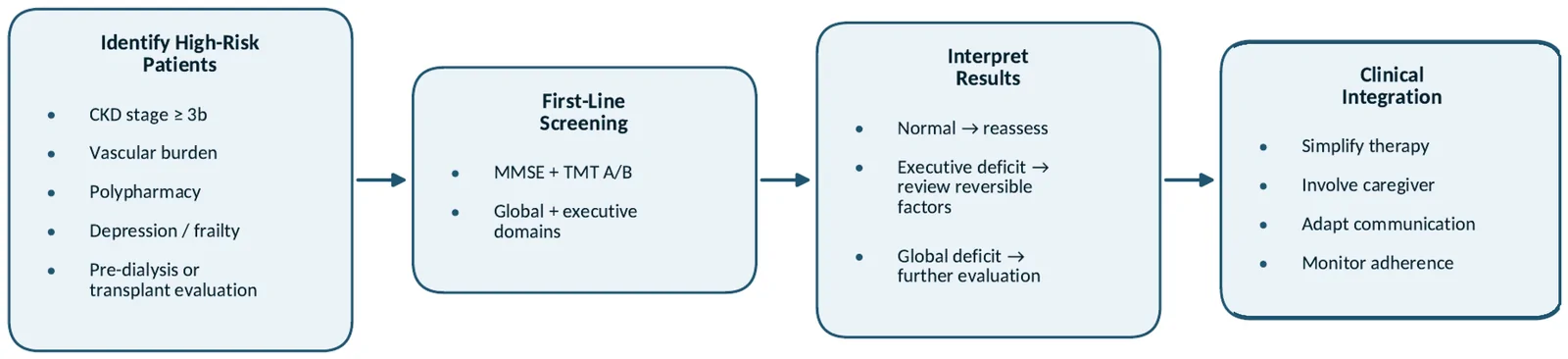
Proposed pragmatic clinical algorithm (author-derived, not prospectively validated) for cognitive screening in CKD. Screening with MMSE and TMT A/B in high-risk patients allows detection of global and executive impairment and guides further evaluation and management.

Practical implementation nonetheless faces real barriers: short visit times, lack of dedicated staff or private space for testing, limited familiarity with cognitive instruments among nephrology staff, and the absence of a standardized workflow for acting on results. Embedding brief screening into existing routine visits (e.g., during dialysis sessions or pre-visit intake), task-sharing with nursing or allied health staff, and integrating screening prompts into electronic health records may help address these barriers, though local validation and staff training remain prerequisites for sustained implementation. The corresponding clinical algorithm is illustrated in Figure 2.

## 6. Clinical Implications

### 6.1. Treatment Adherence and Self-Management

Cognitive impairment in CKD directly affects treatment adherence and self-management. Even mild executive dysfunction—impaired planning, reduced flexibility, slowed processing speed—may compromise medication adherence, dietary compliance, and fluid restriction [10,17]. CKD care requires sustained executive functioning, including coordination of appointments and adherence to complex multidrug regimens. Executive deficits documented in predialysis and dialysis populations [10,17] may therefore contribute to suboptimal metabolic control and inconsistent therapy use.

This adherence burden is compounded in the many CKD patients who also live with multimorbidity and polypharmacy. Multimorbidity, polypharmacy, frailty, and cognitive impairment frequently coexist in older adults with CKD, particularly among the growing population initiating dialysis at advanced age [32]. In this context, medication burden is not fully captured by simple pill counts: potentially inappropriate medications and cumulative anticholinergic load—both common in older CKD patients—have been independently associated with cognitive impairment, falls, and unplanned hospitalizations [33]. For a patient with executive dysfunction, a regimen of ten or more medications spanning several specialists, each with different dosing schedules and renal-adjusted instructions, poses self-management demands that may exceed compensatory capacity, creating a reinforcing cycle in which cognitive impairment undermines adherence, and resulting poor disease control and adverse drug events may in turn further compromise cognitive and physical reserve. Addressing this cycle requires more than patient-directed education: interdisciplinary coordination among nephrologists, primary care providers, and pharmacists—including structured medication reviews—has been associated with improved adherence and reduced polypharmacy-related adverse outcomes in older adults with CKD, and may be particularly valuable for patients in whom cognitive screening has identified executive vulnerability.

In dialysis populations, cognitive impairment is independently associated with adverse outcomes, including mortality [6], indicating that cognitive vulnerability influences clinical trajectory. Identification of impairment should prompt simplified regimens, written instructions, caregiver involvement, and reinforced education [34]. Executive-sensitive measures such as TMT may be particularly informative given their relevance to real-world self-management demands [10].

### 6.2. Decision-Making and Dialysis Initiation

Cognitive impairment is common in advanced CKD and may compromise decision-making capacity when discussing dialysis initiation, modality choice, or conservative management [10,17]. Executive dysfunction can impair risk–benefit evaluation even when global cognition appears preserved [10].

Cognitive assessment in nephrology should therefore identify impaired decision-making capacity in high-stakes contexts [34]. Combined MMSE and executive testing (e.g., TMT A/B) can help flag patients requiring further evaluation [10,29]. Because cognition may fluctuate during acute illness or metabolic instability, reassessment may be necessary before irreversible decisions are made [34]. Caregiver involvement or formal capacity evaluation may be required in selected cases.

### 6.3. Kidney Transplantation

Cognitive impairment influences transplant access and outcomes. Executive dysfunction may limit navigation of evaluation processes and adherence to pre-transplant requirements [10,17]. Impairment has been associated with reduced likelihood of waitlisting [15].

Post-transplant management requires sustained executive functioning for adherence to immunosuppressive therapy and monitoring [34]. Cognitive impairment should not be an automatic exclusion criterion; rather, structured assessment allows identification of patients who require enhanced support and closer follow-up [34].

### 6.4. Hospitalization

Patients with CKD and baseline cognitive impairment are particularly vulnerable during acute illness. Reduced cognitive reserve predisposes to delirium, functional decline, and prolonged recovery [34]. Distinguishing chronic impairment from superimposed delirium is essential, as metabolic disturbances, infections, intradialytic hypotension, and polypharmacy may cause transient fluctuations [34].

Baseline outpatient cognitive assessment facilitates early recognition of delirium and appropriate intervention [34]. Cognitive vulnerability should thus be considered a modifier of acute risk, informing preventive strategies and medication review during hospitalization.

### 6.5. Prognosis and Advance Care Planning

Cognitive impairment in CKD is consistently associated with hospitalization and mortality [2,6] and often parallels functional decline and frailty [29,30]. Cognitive status should therefore inform advance care planning discussions while decision-making capacity remains preserved [34].

Impairment does not imply therapeutic futility; rather, it defines the level of support required and shapes shared decision-making processes [34]. Integrating cognitive assessment into CKD care refines risk stratification and supports ethically grounded clinical planning.

### 6.6. Role of Medical Nutrition Therapy

Medical nutrition therapy may represent a modifiable component of cognitive-risk management in CKD, although direct evidence from CKD-specific intervention trials remains limited. Interest in this approach is supported by the growing recognition that dietary quality may influence brain health through vascular, metabolic, inflammatory, and gut-derived pathways, while CKD adds disease-specific factors such as protein-energy wasting, altered micronutrient status, and the accumulation of gut-derived uremic toxins [35].

Observational studies in the general population have linked healthier dietary patterns and selected food groups with better cognitive outcomes. Higher vegetable and fruit intake has been associated with slower age-related cognitive decline [36], and greater adherence to the Mediterranean-DASH Intervention for Neurodegenerative Delay (MIND) diet has been associated with greater cognitive resilience despite neuropathologic burden [37] and with lower Alzheimer disease pathology [38]. These findings are biologically plausible but should not be extrapolated directly to CKD, in which dietary prescriptions must also account for kidney function, potassium and phosphorus handling, protein requirements, and the risk of malnutrition.

Evidence for individual nutrients is less consistent. Vitamin B12 deficiency is clinically relevant to neurologic function, but supplementation in older adults with moderate deficiency did not improve cognitive outcomes in a randomized trial [39]. In a cross-sectional Chinese study of adults younger than 65 years, a dietary pattern characterized by a higher proportion of energy from fat and protein and a lower proportion from carbohydrate was associated with a higher prevalence of mild cognitive impairment [40]. Polyunsaturated fatty acids and their metabolites have important roles in neurotransmission, neuronal survival, neuroinflammation, and cognition, providing a mechanistic rationale for studying dietary omega-3 intake in cognitive health [41].

The gut–kidney–brain axis provides an additional rationale for nutritional intervention in CKD. Diet shapes the intestinal microbiome and the production of microbial metabolites, including short-chain fatty acids, tryptophan metabolites, and other signaling molecules capable of influencing immune and neural pathways [42]. In CKD, dysbiosis and increased generation or reduced clearance of gut-derived uremic toxins may further amplify neuroinflammation and blood–brain barrier dysfunction [35,43]. Thus, dietary strategies that improve overall diet quality and favorably modify the microbiome are biologically attractive, but their cognitive effects in CKD remain insufficiently tested.

Supplemented very-low-protein diets (sVLPD) with ketoanalogues offer a CKD-specific mechanistic rationale for cognitive protection. By substantially reducing dietary protein-derived nitrogenous load while maintaining essential amino acid supply, sVLPD regimens have been shown to lower serum concentrations of gut microbiota-derived, protein-bound uremic toxins—particularly indoxyl sulfate and *p*-cresyl sulfate—in patients with moderate-to-advanced CKD [44]. These toxins are of particular neurological interest: indoxyl sulfate has been shown, in both animal models and early human imaging studies, to disrupt blood–brain barrier integrity via aryl hydrocarbon receptor activation and to promote neuroinflammation, with associated cognitive impairment [45]. Whether reducing circulating indoxyl sulfate and *p*-cresyl sulfate through sVLPD-based strategies translates into measurable cognitive benefit in CKD has not yet been tested in dedicated interventional trials, but the underlying gut–kidney–brain mechanistic pathway provides a biologically coherent rationale for future study.

A related clinical challenge is balancing dietary phosphorus and potassium restriction—both standard components of CKD nutritional management—against the risk of inadvertently limiting intake of neuroprotective micronutrients, including B vitamins, antioxidants, and polyunsaturated fatty acids, many of which are concentrated in fruits, vegetables, legumes, and fish that are also sources of potassium and phosphorus. Overly restrictive diets pursued without individualized dietary counseling may therefore unintentionally compromise micronutrient adequacy relevant to brain health, even while achieving mineral control. Individualized, dietitian-guided planning that accommodates both mineral restriction and micronutrient sufficiency is consequently a reasonable, if not yet formally tested, target for reducing nutritional contributors to cognitive vulnerability in CKD.

Protein-energy wasting (PEW) and sarcopenia represent a further, potentially modifiable nutritional pathway to cognitive impairment in CKD. PEW—characterized by depleted protein and energy stores alongside chronic inflammation—affects an estimated 10–50% of CKD patients, with prevalence increasing as kidney function declines, and is closely linked to sarcopenia, frailty, and excess mortality. A direct association between PEW and cognitive impairment has been demonstrated in maintenance hemodialysis patients, in whom those with PEW show significantly lower cognitive scores, including in executive function, orientation, and delayed recall domains, than those without PEW [46]. Proposed mechanisms include PEW-related chronic inflammation, impaired neurotransmitter synthesis, and disrupted muscle-brain signaling, though the cross-sectional nature of existing data precludes firm causal conclusions. Given that PEW and sarcopenia are already routine targets of nutritional CKD care, their prevention and treatment represent a practical, low-additional-burden avenue for addressing a nutritional contributor to cognitive vulnerability, pending confirmatory longitudinal and interventional data. These nutritional interventions and their current level of evidence are summarized in Table 2.

**Table 2:** Summary of nutritional interventions and factors relevant to cognitive vulnerability in CKD.

Intervention/Factor	Proposed Mechanism	Current Level of Evidence	Relevance for CKD
sVLPD + ketoanalogues	↓ generation of gut-derived uremic toxins (indoxyl sulfate, *p*-cresyl sulfate), disrupting BBB integrity and promoting neuroinflammation [44,45]	Trials show reduced toxin levels with sVLPD + KA [44]; BBB/cognitive link shown in animal and early human imaging studies [45]; no dedicated RCT with cognitive endpoints in CKD	High relevance—CKD-specific pathophysiology; requires prospective testing with neurocognitive outcomes
Plant-predominant diet	↓ protein-bound uremic toxin precursors; favorable microbiome shift	Observational (general population); limited CKD-specific cognitive data	Plausible, consistent with existing CKD dietary guidance
MIND/Mediterranean-DASH diet	Antioxidant, anti-inflammatory, vascular protection	RCT in general population: no significant cognitive benefit at 3 years [47]; cohort data show cognitive resilience association [37,38]	Extrapolation to CKD limited by dietary constraints (K, P, protein)
B-vitamin correction (e.g., B12)	Homocysteine lowering, neurologic support	RCT in older adults: no significant cognitive benefit from supplementation alone [39]	Relevant for correcting deficiency, not established as cognitive-prevention strategy
Omega-3/polyunsaturated fatty acids	Neurotransmission, anti-neuroinflammatory effects	Mechanistic rationale [41]; limited direct clinical trial data in CKD	Biologically plausible; dedicated CKD trials lacking
PEW/sarcopenia prevention	Systemic catabolic/inflammatory burden, muscle-brain signaling disruption	Direct clinical association between PEW and lower cognitive scores in MHD [46]	High relevance—PEW common (10–50%) and modifiable in CKD
Phosphorus/potassium restriction balanced with micronutrient adequacy	Avoids restrictive diets causing micronutrient deficiency while controlling mineral burden	Clinical/nutritional consensus; not tested specifically for cognitive outcomes	Practical relevance for individualized CKD dietary prescription

Note: ↓ denotes a decrease or reduction in the corresponding parameter.

Importantly, observational associations have not consistently translated into benefit in randomized trials. In the 3-year MIND diet trial involving cognitively unimpaired older adults with a family history of dementia, changes in global cognition and brain MRI measures did not differ significantly between the intervention and control groups [47]. Although this trial was conducted outside the CKD population, it illustrates the limits of inferring treatment effects from observational nutritional data.

For CKD specifically, medical nutrition therapy should therefore be viewed as a plausible but not yet established cognitive intervention. Plant-predominant dietary patterns, individualized protein intake, prevention and treatment of protein-energy wasting, correction of clinically relevant nutrient deficiencies, and strategies aimed at reducing gut-derived uremic toxin generation may all be relevant targets [43,48]. The cognitive effects of low-protein diets, ketoanalogue supplementation, and interventions that modify intestinal toxin production or absorption require dedicated CKD trials with prespecified neurocognitive outcomes before they can be recommended specifically for preservation of cognition [35,43,48].

### 6.7. Economic Considerations

From a health-economic perspective, brief cognitive screening (e.g., MMSE plus TMT A/B) carries low direct costs relative to its potential downstream value: identifying impairment early may reduce avoidable hospitalizations, treatment non-adherence, and complications related to unrecognized decision-making difficulties around dialysis initiation or modality choice. Conversely, unaddressed cognitive impairment has been linked to increased healthcare utilization and mortality, suggesting that the indirect costs of not screening may outweigh the modest resource investment required for routine assessment. Dedicated cost-effectiveness analyses of cognitive screening programs specifically in CKD and dialysis populations are currently lacking and represent an important area for future health-economic research.

## 7. Future Directions

Despite growing recognition of cognitive impairment in CKD, several priorities remain.

First, longitudinal characterization across the CKD trajectory is incomplete. Although prevalence data are robust, longitudinal characterization across the full CKD trajectory—from earlier CKD through dialysis and transplantation—remains limited [49]. Clarifying whether decline follows linear, threshold-dependent, or accelerated patterns would improve timing of screening and intervention.

Second, standardization of cognitive assessment is needed. Instrument heterogeneity limits comparability across studies [10]. A consensus-driven minimal screening battery—balancing feasibility and executive-domain sensitivity—would enhance reproducibility and clinical integration. Defined reassessment intervals and clinically meaningful thresholds for change also require development.

Third, interventional research targeting modifiable contributors is essential. Optimization of blood pressure, glycemic control, and anemia management may influence cognitive trajectories. Dialysis-related hemodynamic instability represents an additional potential target [34]. Future interventional studies should also incorporate rigorous methodological design and appropriate statistical approaches, while accounting for the ethical and regulatory challenges inherent to comparative clinical research [50].

Future interventional trials should also consider incorporating nutritional strategies—such as individualized protein prescription, correction of micronutrient deficiencies, and interventions targeting gut-derived uremic toxin generation—as adjunctive or standalone arms, with prespecified neurocognitive endpoints, to build the currently limited evidence base for medical nutrition therapy as a cognitive-risk intervention in CKD.

Finally, the prognostic significance of cognitive impairment—particularly its association with hospitalization and mortality in CKD populations [6]—raises important ethical and operational questions regarding advance care planning, dialysis decisions and transplant eligibility [34]. New studies are necessary to clarify how cognitive findings can guide supportive strategies without exacerbating inequities.

Advancement in this field will require coordinated, multidisciplinary efforts aimed not only at detection, but at prevention and trajectory modification.

### Limitations

This review has several limitations. As a narrative rather than a systematic review, it does not follow a pre-registered protocol or formal risk-of-bias assessment, and study selection—while thematically guided—retains an element of author judgment, introducing potential selection bias. The literature synthesized is heterogeneous in study populations (predialysis CKD, hemodialysis, peritoneal dialysis, and post-transplant), cognitive assessment instruments (ranging from brief screens such as the MMSE to detailed neuropsychological batteries), and definitions of cognitive impairment, limiting direct comparability across studies. Differences between CKD stages and renal replacement modalities may also affect the generalizability of specific findings, as illustrated by the heterogeneous evidence on cognitive outcomes by dialysis modality discussed in Section 3.6. These limitations should be considered when interpreting the synthesized evidence and the proposed screening algorithm.

## 8. Conclusions

Cognitive impairment is increasingly recognized as a frequent and clinically meaningful component of chronic kidney disease (CKD). Across the disease spectrum—from early CKD to dialysis and transplantation—cognitive vulnerability reflects cumulative vascular burden, metabolic stress, and reduced cerebral reserve.

The prevailing cognitive phenotype in CKD is dominated by executive dysfunction and slowed processing speed, domains that directly affect self-management, adherence, and complex medical decision-making. Cognitive impairment should therefore be viewed not as an incidental comorbidity, but as a modifier of clinical trajectory.

Pragmatic screening strategies combining global and executive-domain assessment can be feasibly integrated into nephrology practice. Early identification enables tailored communication, structured decision-making support, and refined risk stratification. In advanced CKD, cognitive status becomes central to ethically sound discussions regarding dialysis initiation, transplantation, and advance care planning.

In practical terms, the evidence reviewed here supports five concrete actions for nephrology practice. First, prioritize brief cognitive screening (MMSE combined with TMT A/B) in patients at elevated risk—CKD stage ≥3b, rapid renal decline, high vascular burden, polypharmacy, depressive symptoms, frailty, and especially before dialysis initiation or transplant evaluation. Second, when testing hemodialysis patients, document (and where feasible standardize) test timing relative to the dialysis session, given the partially reversible, session-related component of cognitive performance. Third, when executive dysfunction is identified, systematically review potentially reversible contributors—anticholinergic medication burden, anemia, depressive symptoms, and metabolic disturbances—before attributing findings to irreversible decline. Fourth, act on confirmed impairment by simplifying medication regimens, providing written and caregiver-supported instructions, and adjusting the pace and format of shared decision-making around dialysis modality, transplantation, and advance care planning. Fifth, integrate nutritional assessment into this pathway — correcting protein-energy wasting and micronutrient deficiencies where present—as a low-burden, clinically reasonable adjunct, while recognizing that dedicated nutritional interventions for cognitive outcomes in CKD remain to be formally tested in prospective trials.

Future progress will require longitudinal characterization, standardized screening frameworks, and interventional strategies targeting modifiable contributors, including nutritional vulnerability. While nutritional strategies are biologically plausible and represent a promising avenue, they remain unproven as cognition-specific interventions in CKD; the transition from screening and early diagnosis to adequately tested therapeutic and nutritional interventions—validated in dedicated CKD trials with prespecified neurocognitive outcomes—represents the next critical step.

Ultimately, integrating cognitive assessment into CKD care reframes kidney disease as a systemic condition affecting brain resilience, autonomy, and therapeutic decisions. Recognizing cognitive vulnerability is essential to defining comprehensive nephrology practice in the modern era.

## Data Availability

No new data were created or analyzed in this study. Data sharing is not applicable to this article.
